# Evaluating Daily Versus Global Stress Appraisals’ Sensitivity to Mild Cognitive Impairment

**DOI:** 10.1093/geroni/igaa057.2014

**Published:** 2020-12-16

**Authors:** Eric Cerino, Stacey Scott, Ruixue Zhaoyang, Richard Lipton, Martin Sliwinski

**Affiliations:** 1 Pennsylvania State University, University Park, Pennsylvania, United States; 2 Stony Brook University, Stony Brook, New York, United States; 3 The Pennsylvania State University, University Park, Pennsylvania, United States; 4 Albert Einstein College of Medicine, Bronx, New York, United States; 5 Penn State University, University Park, Pennsylvania, United States

## Abstract

Stress is an important correlate of cognitive aging that manifests in everyday life. Infrequent trait-based stress measures may not be as sensitive to mild cognitive impairment (MCI) as ecological momentary assessments (EMA). We compared EMA to global trait-based stress measures in discriminating MCI. A sample of 248 adults from the Einstein Aging Study (Mage=77.33 years, SD=5.04; 68 with MCI) were prompted to report whether a stressor occurred and to rate the severity up to four times daily for 14 days. Global perceived stress and neuroticism were assessed at baseline. Although MCI status was unrelated to stressor frequency (p>.05), individuals with MCI appraised their daily stressors as more severe than cognitively intact participants (p=.03). No MCI-related differences emerged on global stress or neuroticism assessments (ps>.05). Results suggest everyday stress markers may be more sensitive to differentiating MCI than global assessments and point toward their utility for early identification of pathological declines.

